# Validation of 3D echocardiographic volume detection of left atrium by human cadaveric casts

**DOI:** 10.1186/s12880-018-0282-4

**Published:** 2018-11-15

**Authors:** Jouni K. Kuusisto, Vesa M. Järvinen, Juha P. Sinisalo

**Affiliations:** 1 Division of Cardiology, Heart and Lung Center, Helsinki University Central Hospital, Meilahti Tower Hospital, P.O. Box 340, FIN-00029 HUS Helsinki, Finland; 2 Department of Clinical Physiology, Medical Imaging Center, Hospital District Helsinki and Uusimaa, Hyvinkää Hospital, Hyvinkää, Finland

**Keywords:** Cardiac imaging, Echocardiography, LAV, 3DE, 2DE, Area-length method, In vitro

## Abstract

**Background:**

Left atrial volume is a prognostic factor in cardiac pathologies. We aimed to validate left atrial volume detection with 3D and 2D echocardiography (3DE and 2DE) by human cadaveric casts. 3DE facilitates measurement of atrial volume without geometrical assumptions or dependence on imaging angle in contrast to 2DE methods.

**Methods:**

For method validation, six water-filled balloons were submerged in a 20-l water tank and their volumes were measured with 3DE. Seven human cadaveric left atrial casts were prepared of silicone and were transformed into ultrasound-permeable casts. Casts were imaged in the same setting, so that 3DE and 2DE of casts represented transthoracic apical view. Left ventricle analysis softwares GE 4D Auto LVQ and TomTec 4D LV-Function were used for 3DE volumetry.

**Results:**

Balloon volumes ranged 37 to 255 ml (mean 126 ml). 3DE resulted in an excellent volumetric agreement with balloon volumes, absolute bias was − 3.7 ml (95% CI -5.9 to − 1.4). Atrial cast volumes were 38 to 94 ml (mean 56.6 ml). 3DE and 2DE volumes were excellently correlated with cast volumes (*r* = 0.96 to 0.99). Biases were for GE 4D LVQ -0.7 ml (95% CI -6.1 to 4.6), TomTec 4D LV-Function 3.3 ml (− 1.9 to 8.5) and 2DE 2.9 ml (− 4.0 to 9.9). 3DE resulted in lower limits of agreement and showed no volume-related bias in contrast to area-length method.

**Conclusions:**

We conclude that measurement of human cadaveric left atrial cast volumes by 3DE is in excellent agreement with true cast volumes.

## Background

Left atrial (LA) volume and depressed function of LA are prognostic factors of adverse cardiovascular events in cardiac pathologies, such as atrial fibrillation, heart failure and coronary artery disease [1–5], and in general population [6]. LA size can be estimated by measuring its diameter, which is associated with cardiac events, but has limited correlation to atrial volume [7]. Calculated LA volumes by two-dimensional echocardiographic (2DE) methods are stronger predictors of adverse outcomes in comparison with LA diameter or LA cross sectional area [8]. 2DE methods are recommended for the volumetric assessment in the current guideline of echocardiographic chamber quantification [9] and in recommendation of cardiac imaging in atrial fibrillation [10]. Still an underestimation of volume by 2DE methods is evident [11, 12]. Three-dimensional echocardiography (3DE) provides measure of LA volume without geometrical assumptions, which are implied in 2DE methods. Agreement of 3DE is shown to be good in comparison to cardiac magnetic resonance imaging, although there is some discrepancy in results, which might be due to various factors i.e. different imaging hardware, imaging settings and analysis algorithms. [13–15] Cardiac magnetic resonance is considered gold standard in left atrial volumetry.

Previously Chen et al. validated in vitro 3DE volume detection by excised porcine hearts for right ventricle and demonstrated superiority of 3DE over 2DE methods [16]. In 2001 Teupe et al. used 3DE to measure normal and aneurysmal left ventricles of excised pig hearts to demonstrate accuracy of 3DE volumetric method [17]. In vitro validation of cardiac MRI volume detection has been demonstrated for both human atria by human cadaveric casts [18, 19].

In this study, we imaged human cadaveric left atrial casts by 3DE and 2DE to test agreement of volumetric methods to true cast volumes measured by water displacement. We also imaged water-filled balloons to test 3DE in wider range of volumes and to validate most appropriate placement of volumetric borderline in measurements.

## Methods

### Materials

Six latex balloons were filled with water to represent volumes of LA both in normal physiological conditions and in left atrial enlargement. The balloons were relatively spherical in shape and their walls provided easily distinguishable ultrasound echo for analysis.

Seven human cadaveric LA casts were prepared in the Department of Forensic Medicine, Helsinki University, Finland. No fixation of cardiac tissues was used. Mitral valves and ventricular apices were removed, and the pulmonary veins were clamped. Hearts were suspended from apical portion and left hearts were filled with silicone rubber without extra filling pressure. After hardening of the silicone rubber, the casts were removed from the hearts. LA parts of the casts were separated from ventricular parts at the mitral annular level. These casts were previously used in magnetic resonance imaging studies by Järvinen and Jauhiainen in the 1990s [18, 19]. At that time, no approval of ethics committee was required and the study was approved by head of department.

**Fig. 1:**
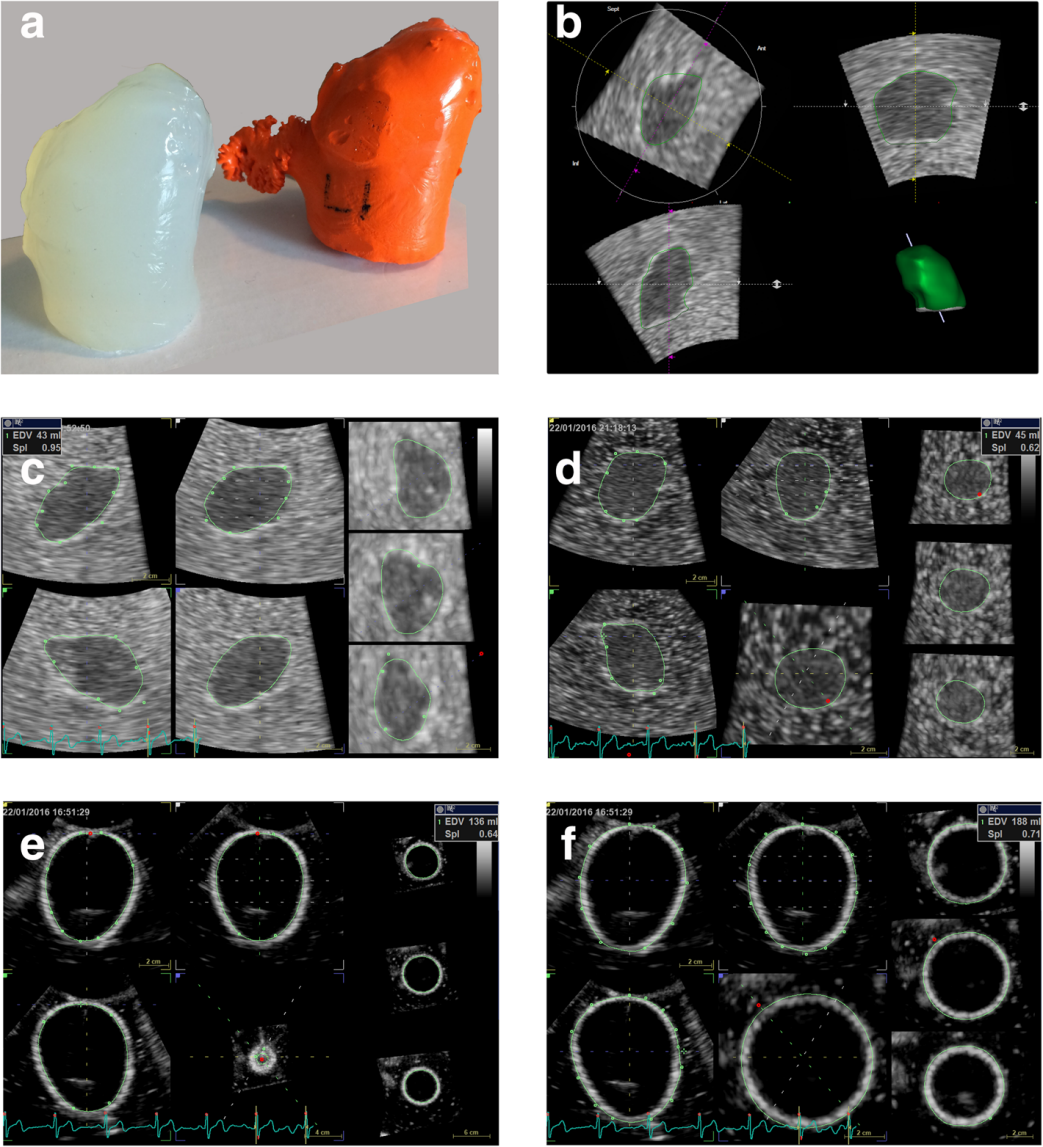
**a**) Human cadaveric left atrial silicone cast (on right) and its transformed agar-agar cast without left atrial appendage, **b**) 3D echocardiographic volume analysis of cast no. 4 by TomTec 4D LV-Function (true volume 43 ml, measured volume 42 ml) and by **c**) GE 4D LVQ (measured volume 43 ml), **d**) cast no. 5 by GE 4d LVQ (true volume 44 ml, measured volume 45 ml), **e**) 3D volume measurement by GE 4D LVQ of a balloon with borderline on the inner aspect (true volume 140 ml, measured volume 136 ml) and **f**) on the outer aspect (measured volume 188 ml)

For this study the silicone casts were transformed to casts made of agar-agar, as casts of silicone rubber are not permeable to ultrasound. Molds for agar-agar casts were made from latex rubber, which was applied on the primary silicone casts while they were stabilized at their mitral planes to a level surface. Four layers of latex rubber was applied on each silicone cast. Left atrial appendices were excluded at this point from the latex molds, as they are usually not included in the volumetric assessment of left atrium by transthoracic ultrasound. The latex molds were peeled from silicone casts and two further layers of latex rubber were used to shut the open left atrial appendix orifices. The latex molds were then positioned so that the mitral openings were facing upwards. A 1,5% agar-agar and water solution was prepared by heating until boiling and then poured into the latex molds at the temperature of 60 degrees centigrade. The molds were cast until the level of mitral annulus. The agar-agar casts were refrigerated overnight and then they were carefully removed from the molds (Fig. 1a).

### Determination of cast and balloon volumes

True volumes of the balloons were determined by weighing the water-filled balloons, assuming one gram of weight representing one milliliter of volume. The weight of the balloons before filling with water were measured to be insignificantly low.

True volumes of the agar-agar casts were determined by volume displacement method. A vessel with an opening on its side was filled with water up to the lower level of the opening. The casts were carefully submerged in to the water and the displaced water was collected through the opening into a 100-ml measuring glass. The volume of the displaced water was assumed to be the true volume of the cast. Volumes were determined by this method before and right after imaging to detect whether the cast volumes had been affected by the immersion into water during imaging.

### Imaging

GE Vivid E9 machine with 4 V probe (GE Vingmed Ultrasound AS, Horten, Norway) was used for both 3DE and 2DE imaging. The agar-agar casts were immersed in a twenty-liter tank for imaging. Water mixture with dried and crushed seeds of *Plantago ovata* was used as the imaging medium, as enhanced contrast of the water was necessary for the semi-automated volumetry softwares to function properly. Higher signal intensity of the medium than that of casts represents the relative signal intensities in the in vivo measurements where signal intensity from myocardium and other surrounding tissues is higher than from blood in the atrial cavity. Coarse cloth was placed on the bottom of the tank to attenuate reverberation. The probe was supported above the tank by a tripod so that lens of the probe was 10 mm below the water surface and orienting downwards.

The casts were stationed on a thin (diameter 3 mm) wooden support attached to a pedestal on the bottom of the tank to stabilize the cast during imaging, and the casts were positioned to represent transthoracic apical view. Mitral annular level of the cast was horizontal and thus perpendicular to ultrasound wave propagation. The orifice of left atrial appendix was positioned 60 degrees counterclockwise from the 2DE view so that the first aspect of the imaging represented the apical four chamber view. The ultrasound beam was then electronically rotated 60 degrees counterclockwise to obtain two chamber view. Zoomed 4D view was used to collect 3DE volumetric data over 4 to 6 cardiac cycles, which were defined from electrocardiogram recorded from researcher at a heat rate of 65 to 70 bpm. The gain was optimized by eye for best possible delineation. Recorded volume size and probe frequency were adjusted so that the volume frame rate was 35 to 50 Hz which is the typical acquisition frequency when imaging dynamic volume in vivo.

Water balloons were stationed by a thread and weight to the bottom of the tank so that the center of the balloons was approximately at 100 mm distance from the lens of the probe. 3DE images were collected similarly. No contrast enhancement agent was used during balloon imaging.

### Image analysis

Image analysis was performed offline with GE EchoPAC work station software version 112.1.1 (GE Vingmed Ultrasound AS, Horten, Norway) after imaging. GE EchoPAC 4D LVQ (Figs. 1c–f) and TomTec 4D LV-Function (TomTec Imaging Systems GmbH, Unterschleissheim, Germany) (Fig. 1b) softwares were used for volume analysis. Both softwares are designed for cardiac left ventricle volume analysis, but they can also be applied for LA volumetry, as we demonstrate in this study. The researcher was blinded to the weights of the balloons and the results of volume displacement representing true volumes of balloons and casts.

There was a distinct border echo in the ultrasound images at the interface of water as medium, balloon wall, and water inside the balloon. This border echo thickness in the images was clearly greater than that of the balloon true walls (< 1 mm). To assess the most appropriate approach of volumetric measurement, the borderline was placed on the inner (Fig. 1e) and outer rims (Fig. 1f), and to the middle of this border echo by GE 4D LVQ. Automation of TomTec software assumed the borderline to the inner aspect of the rim, so only this approach was used by TomTec software.

Measurements from the ultrasound images representing apical view during cast imaging were 1) 3DE volumetry by both softwares, 2) LA cast cross section areas from four and two chamber views and 3) the greatest length of these cross sections from middle of mitral orifice area to the roof of the atrial casts. The four and two chamber areas and their respective lengths were used to calculate approximations of volumes by biplane area-length method (A-L method) by equation $\frac{8}{3\pi}\times \frac{4 ch\_ area\times 2 ch\_ area}{length}$, where length is the shorter of the two measured lengths. Repeatability was tested for 3DE volumetry and A-L method of left atrial casts by a time interval of two weeks between repeated measurements.

### Statistical and data analyses

IBM SPSS for Macintosh version 24.0 (Armonk, NY, USA) and Microsoft Excel for Mac version 15.26 (Microsoft Corporation, Redmond, WA, USA) were used for statistical and data analyses. Mean and range of true and measured volumes of balloons were determined. Paired differences of measured volumes to true volumes were calculated. Mean of these differences were considered bias. 95% confidence intervals (95% CI) for bias and limits of agreement (LOA), defined as bias ±1.96 standard deviations, were calculated. LOA represent the range in which 95% of measured volumes differ from true volumes when normal distribution is assumed. Pearson correlation coefficients and their statistical significances were calculated. The same statistical methods were applied for repeated volumetric measurements in addition to intraclass correlation coefficients and their statistical significance. Two-way mixed testing for absolute agreement was used for intraclass correlation. Bland-Altman difference plots were used to visualize data.

## Results

### Balloon imaging

True volumes of the water balloons ranged from 37 to 255 ml and mean volume was 126 ml. All the tested 3DE volumetric methods, including all borderline placements by GE 4D LVQ, to measure balloon volumes resulted in very high correlations to true volumes (Pearson *r* > 0.999, *p* < 0.0005). Balloon wall echo thickness was 1 to 6 mm and it was lowest in areas where ultrasound wave propagation was perpendicular to the wall balloon wall. Placement of the borderline on the inner aspect of the balloon wall echo by GE 4D LVQ resulted lowest absolute bias − 3.7 ml (95% CI -5.9 to − 1.4) and the bias did not correlate to volume (Pearson *r* = − 0.36, *p* = 0.481). LOA were − 7.9 to 0.6 ml. Placement of borderline to the midline or outer aspect of the wall echo produced volume dependent overestimation of the volume and the biases were 15.5 ml (95% CI 4.7 to 26.3) and 44.2 ml (11.4 to 77.0), respectively. With TomTec software the bias was inversely related to volume resulting in greater negative bias in larger observed volumes. Mean bias was − 24,3 ml (95% CI -37.8 to − 10.8) with TomTec. As noted previously, semi-automation in TomTec software prevented precise manual adjustments to the borderline.

### Cast imaging

Agar-agar cast volumes determined by volume displacement ranged from 38 to 94 ml (mean 56.6 ml). Repeated volume displacement measurements before and after imaging resulted in maximum of 2 ml difference. The measured volume displacement before the imaging was used in the later analyses.

**Fig. 2:**
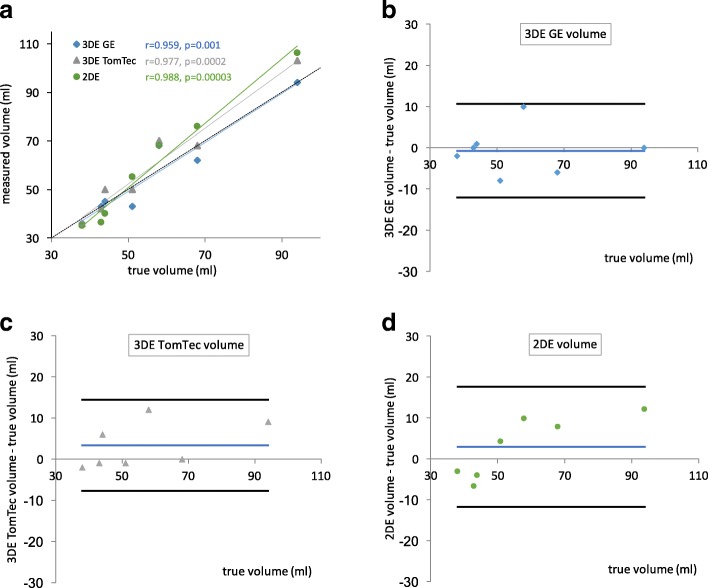
Results of human left atrial cast volume measurements by 3DE and 2DE. **a**) 3DE and 2DE volumes in comparison to true volumes of casts. **b**-**d**) Bland-Altman paired difference plots of volumetric measurements in comparison to true volumes. Black lines represent limits of agreement (mean ± 1.96 SD) and blue lines bias. 2DE volume – volume calculated by 2D echocardiographic biplane area-length method, 3DE GE and 3DE TomTec – 3D echocardiographic volume measured by GE 4D LVQ and TomTec 4D LV-Function softwares, respectively, r – Pearson correlation coefficient.

The interface with the medium and agar-agar left atrial casts produced less prominent wall echo than with balloon imaging. As suggested most appropriate by balloon measurements, the borderline was placed closely to inner aspect of the echo representing interface of the cast and medium during 3DE volumetry and A-L method. Pearson correlation coefficients of measured volumes to true volumes for all methods were high and 95% CI of biases for these methods included zero bias. 3DE volumetric measurements with both softwares resulted in narrower limits of agreement in comparison to A-L method. With A-L method bias was positively correlated with volume (Pearson r of difference to true volume was 0.837 (*p* < 0.05)), but no statistically significant volume dependent bias was observed in 3DE methods (GE 4d LVQ *p* = 0.96 and TomTec 4d LV-Function *p* = 0.24). Measured volumes for casts, their displaced volumes and statistic characteristics are presented in Table 1 and Fig. 2a. Bland-Altman difference plots visualize the observed differences, biases and limits of agreement (Figs. 2b-d).

**Table 1:** 3DE volumetric measurements and 2DE calculated volumes of left atrial human cadaveric casts in comparison to their true volumes

		3DE volume: GE 4D LVQ		3DE volume: TomTec LV		2DE volume	
cast no.	true volume (ml)	measured volume (ml)	difference to true volume (ml)	measured volume (ml)	difference to true volume (ml)	calculated volume (ml)	difference to true volume (ml)
1	38	36	−2	36	-2	35	−3
2	94	94	0	103	9	106	12
3	51	43	−8	50	−1	55	4
4	43	43	0	42	− 1	36	−7
5	44	45	1	50	6	40	−4
6	58	68	10	70	12	68	10
7	68	62	−6	68	0	76	8
mean (95% CI)	56.6	55.9	−0.7 (−6.1–4.6)	59.9	3.3 (−1.9–8.5)	59.5	2.9 (−4.0–9.9)
SD	19.4	20.3	5.8	22.8	5.6	26.0	7.5
range	38–94	36–94	−8–10	36–103	−2–12	35–106	−7–12
LOA			−12.1–10.6		−7.8–14.4		−11.9–17.6
r		0.959 (*p* = 0.001)		0.977 (*p* = 0.0002)		0.988 (*p* = 0.00003)	

95% CI – 95% confidence interval, 2DE volume – volume calculated by 2D echocardiographic biplane area-length method, 3DE volume: GE 4D LVQ and 3DE volume: TomTec LV – 3D echocardiographic volume measured by GE 4D LVQ and TomTec 4D LV-Function softwares, respectively, LOA – limits of agreement defined as mean ± 1.96 SD, r – Pearson correlation coefficient, SD – standard deviation

### Repeatability

**Fig. 3:**
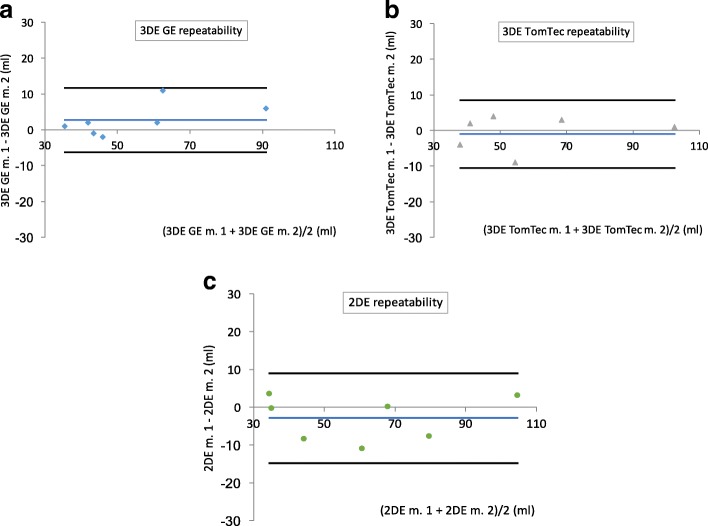
Results of repeated measurements of human left atrial cast volumes. Bland-Altman paired difference plots of repeated volumetric measurements of casts in comparison to mean of measured volumes. Black lines represent limits of agreement (mean ± 1.96 SD) and blue lines mean of differences to paired averages of measured volumes. **a** 3DE GE repeatability - 3D echocardiographic repeated volume measurements by GE 4D LVQ software, **b** 3DE TomTec repeatability - 3D echocardiographic repeated volume measurements by TomTec 4D LV-Function software, **c** 2DE repeatability – repeated calculated volumes by biplane area-length method, m. 1 and m. 2 – first and repeated measurement

Repeated 3DE and A-L method volumetric measurements were performed to test repeatability. Both Pearson and intraclass correlation coefficients indicated very good repeatability (*r* = 0.975 to 0.982, intraclass correlation coefficients 0.967 to 0.980). Results of these measurements are depicted in Table 2. Bland-Altman charts describe the relation of measurement differences to average volumes of measurements (Figs. 3a–c).

**Table 2:** Repeated 3D volumetric measurements and 2D calculated volumes of left atrial human cadaveric casts

cast no.	3DE volume: GE 4D LVQ			3DE volume: TomTec LV			2DE volume		
	meas. 1 (ml)	meas. 2 (ml)	difference (ml)	meas. 1 (ml)	meas. 2 (ml)	difference (ml)	meas. 1 (ml)	meas. 2 (ml)	difference (ml)
1	36	35	−1	36	40	4	35	35	0
2	94	88	−6	103	102	−1	106	103	−3
3	43	44	1	50	59	9	55	66	11
4	43	41	−2	42	40	−2	36	33	−3
5	45	47	2	50	46	−4	40	48	8
6	68	57	−11	70	67	−3	68	68	0
7	62	60	−2	68	72	4	76	84	8
mean (95% CI)	55.9	53.1	−2.7 (−6.8–1.4)	59.9	60.9	1.0 (−3.4–5.4)	59.4	62.4	3.0 (−2.4–8.4)
SD	20.3	17.7	4.5	22.8	22.2	4.8	26.0	25.8	5.8
range	36–94	35–88	−11–2	36–103	40–102	−4–9	35–106	33–103	−3–11
LOA			−11.5–6.0			−8.3–10.3			−8.4–14.4
r	0.982, *p* = 0.0001			0.978, p = 0.0001			0.975, p = 0.0002		
ICC (95% CI)	0.967 (0.819–0.994), p = 0.00003			0.980 (0.894–0.996), *p* = 0.00001			0.972 (0.857–0.995), *p* = 0.00002		

95% CI – 95% confidence interval, 2DE volume – volume calculated by 2D echocardiographic biplane area-length method, 3DE volume: GE 4D LVQ and 3DE volume: TomTec LV – 3D echocardiographic volume measured by GE 4D LVQ and TomTec 4D LV-Function softwares, respectively, ICC – intraclass correlation coefficient, LOA – limits of agreement defined as mean ± 1.96 SD, meas. 1 and meas. 2 ¬ first and repeated measurement, r – Pearson correlation coefficient for meas. 1 and meas. 2, SD – standard deviation

## Discussion

In this study we tested the applicability of 3D and 2D echocardiographic volumetric methods in determining volumes of human left atrial casts. To our knowledge validation of left atrial volumetry in this setting has not been done previously. We showed that 3DE volumetry of water-filled balloons is in excellent absolute agreement with true volumes of the balloons when volumetric borderlines were placed closely on the inner aspect of the balloon wall echo. We found 3DE methods to be equally or more accurate in comparison to 2DE represented by biplane area-length method, considering lower observed limits of agreement and lack of volume dependent bias with 3DE. All methods had excellent correlation to true cast volumes and were well repeatable.

With 2DE volumetric methods there are inherent assumptions of geometrical symmetricity. With biplane area-length method an ellipsoid, and with monoplane area-length a spheroid shape is assumed. In addition to this geometrical assumption, the in vivo transthoracic apical views that are oriented along the left ventricular axis might not represent the left atrial axis. The planimetered area and length of the left atrium can thus be a misestimation resulting in an unpredictable error in estimation of the atrial volume [20]. Underestimation of volume by A-L method in comparison to cardiac magnetic resonance imaging is reported [21, 22]. With 3DE volumetry there is no need for geometrical assumptions or for orienting the echocardiographic beam along the left ventricular or atrial axis as the volume is measured along the true borders of the chamber.

Casting process and post mortem state might have affected volumes and shapes of the casts, and possible prior medical conditions of the deceased are not known. For the scope of this study we did not consider this a limitation as we aimed for volumetric validation of casts that represent the variable sizes and shapes of human left atria. Regarding some casts, areas of the interface of the cast and the medium were not explicit which at least partly contributed both to error in volumetric measurements to true volumes and repeatability. Different choice of medium or cast material could have mitigated this issue. In vitro repeatability in this study is likely better in comparison to clinical setting which has more variability in the imaging conditions.

The walls of water-filled balloons produced distinct echo lining in the images which we speculate to be at least partly due to interference of the transmitted ultrasound signal and the balloon wall. In cast imaging the interface consists of transition from contrast enhanced water to cast as a medium for ultrasound propagation which probably induces less artifacts in comparison to balloon wall. We noted, however, a less pronounced lining in the images also during cast imaging. The contrast enhanced water tended to turn less homogenous over time and stirring of the medium was required intermittently. We speculate this lining during cast imaging to consist mostly of the contrast enhanced medium, not the cast itself, giving a rationale for the placement of the volumetric borderline to inner aspect of the interface.

## Conclusions

We conclude that 3DE is an accurate and feasible method to image left atrial volume in this in vitro study. Commercially available software (GE 4D LVQ and TomTec 4D LV-Function) developed for the left ventricular volume analysis can be used in the left atrial volume analysis as well.

## Abbreviations

2DE: Two-dimensional echocardiography
3DE: Three-dimensional echocardiography
95% CI: 95% confidence interval
A-L Method: Biplane area-length method
LA: Left atrium
LOA: Limits of agreement
